# Recommendations for diabetic macular edema management by retina specialists and large language model-based artificial intelligence platforms

**DOI:** 10.1186/s40942-024-00544-6

**Published:** 2024-02-28

**Authors:** Ayushi Choudhary, Nikhil Gopalakrishnan, Aishwarya Joshi, Divya Balakrishnan, Jay Chhablani, Naresh Kumar Yadav, Nikitha Gurram Reddy, Padmaja Kumari Rani, Priyanka Gandhi, Rohit Shetty, Rupak Roy, Snehal Bavaskar, Vishma Prabhu, Ramesh Venkatesh

**Affiliations:** 1 Dept. of Retina and Vitreous, Narayana Nethralaya, #121/C, 1st R Block, Chord Road, Rajaji Nagar, 560010 Bengaluru, Karnataka India; 2 Dept of Retina and Vitreous, Little Flower Hospital and Research Centre, 683572 Angamaly, Kerala India; 3 Medical Retina and Vitreoretinal Surgery, University of Pittsburgh School of Medicine, 203 Lothrop Street, Suite 800, 15213 Pittsburg, PA USA; 4 Anant Bajaj Retina Institute, L V Prasad Eye Institute, Kallam Anji Reddy Campus, 500034 Hyderabad, Telangana India; 5 Dept. of Cornea and Refractive Services, Narayana Nethralaya, #121/C, 1st R Block, Chord Road, Rajaji Nagar, 560010 Bengaluru, Karnataka India; 6 Dept. of Vitreo-Retina, Aditya Birla Sankara Nethralaya, 700099 Kolkata, India

**Keywords:** Diabetic macular edema, Management, Guidelines, Diabetic retinopathy, Artificial intelligence

## Abstract

**Purpose:**

To study the role of artificial intelligence (AI) in developing diabetic macular edema (DME) management recommendations by creating and comparing responses to clinicians in hypothetical AI-generated case scenarios. The study also examined whether its joint recommendations followed national DME management guidelines.

**Methods:**

The AI hypothetically generated 50 ocular case scenarios from 25 patients using keywords like age, gender, type, duration and control of diabetes, visual acuity, lens status, retinopathy stage, coexisting ocular and systemic co-morbidities, and DME-related retinal imaging findings. For DME and ocular co-morbidity management, we calculated inter-rater agreements (kappa analysis) separately for clinician responses, AI-platforms, and the “majority clinician response” (the maximum number of identical clinician responses) and “majority AI-platform” (the maximum number of identical AI responses). Treatment recommendations for various situations were compared to the Indian national guidelines.

**Results:**

For DME management, clinicians (ĸ=0.6), AI platforms (ĸ=0.58), and the ‘majority clinician response’ and ‘majority AI response’ (ĸ=0.69) had moderate to substantial inter-rate agreement. The study showed fair to substantial agreement for ocular co-morbidity management between clinicians (ĸ=0.8), AI platforms (ĸ=0.36), and the ‘majority clinician response’ and ‘majority AI response’ (ĸ=0.49). Many of the current study’s recommendations and national clinical guidelines agreed and disagreed. When treating center-involving DME with very good visual acuity, lattice degeneration, renal disease, anaemia, and a recent history of cardiovascular disease, there were clear disagreements.

**Conclusion:**

For the first time, this study recommends DME management using large language model-based generative AI. The study’s findings could guide in revising the global DME management guidelines.

**Supplementary Information:**

The online version contains supplementary material available at 10.1186/s40942-024-00544-6.

## Introduction

Diabetic retinopathy (DR) is one of the many serious eye complications associated with diabetes mellitus [1, 2]. Diabetic macular edema (DME) is one of two reasons for sight-threatening DR, the other being proliferative DR. In India, the prevalence of DME is less than 10% (range: 2.4 − 8.9%) [3–6]. The absolute number of people with DME in India is significant due to the rising number of diabetics. Various treatment modalities exist for the management of DME, and the selection of treatment depends primarily on the availability of retinal specialists and treatment facilities, as well as the patient’s economic status and underlying ocular and systemic conditions [7]. Because the prevalence of diabetes mellitus, and thus DR and DME, varies across the globe, several countries and regions have developed their own independent guidelines for the screening and management of DR and DME [8–12]. Similarly, the All-India Ophthalmology Society (AIOS) and the Vitreo-Retinal Society of India (VRSI) [national guidelines] have collaborated to develop a consensus statement on the practice points of DME management in India, with the objective of describing the preferred practice patterns for DME management in different clinical situations [13]. 

Several discussions have centered on the potential advantages and disadvantages of incorporating artificial intelligence (AI) into medicine, including ophthalmology. To date, several papers have been published using deep machine learning-based algorithms to identify and guide DME treatment using color fundus photographs and optical coherence tomography (OCT) images [14–17]. Concerns have been raised about data acquisition, data bias, identifying ground truth, comparing different algorithms, machine learning challenges, its application to different groups of people, and human barriers to AI adoption in health care [18]. A large language model (LLM) or natural language processing is a form of generative AI algorithm that understands, summarizes, generates, and predicts new text-based content using deep learning techniques and massively large data sets [19]. Many such open source LLM-based generative AI algorithms are currently freely and easily available, including OpenAI’s ChatGPT3.5v and ChatGPT4.0v, BARD from Google, Bing AI from Microsoft, and others [20]. Most researchers and clinicians believe that AIs based on LLM, when integrated into the electronic health record, could aid in the development of the best DME treatment strategy [21]. To the best of our knowledge, we could not find any literature that explored the role of LLM-based AI in DME management.

Thus, the primary objective of this study was to investigate the role of AI in formulating treatment recommendations for DME management by generating and comparing its responses to those of clinicians in different AI-generated clinical case situations. The authors also intended to compare the recommendations obtained collectively by clinicians and different AI platforms to the national guidelines for DME management for the case situations described in this study.

## Methods

This was a prospectively conducted questionnaire-based study. The first stage of the study began by asking ChatGPT 3.5v (OpenAI, San Francisco, CA, USA) to generate 25 hypothetical clinical cases involving diabetes mellitus and providing information about the DR and DME status for each eye separately. This was accomplished by instructing the ChatGPT to use the pointers listed below while creating the clinical case. The following pointers were included: (1) patient demographics - age and gender; (2) diabetes type, duration, and control; (3) recent onset visual symptoms; (4) visual acuity (VA) of both eyes in Snellen’s format; (5) lens status - whether clear lens, cataractous lens, pseudophakia, or aphakia; (6) clinical fundus findings description - include cases of both non-proliferative and proliferative disease; (7) presence of systemic co-morbidities such as renal disease, hypertension, anemia, cardiovascular disease, and a deranged lipid profile; (8) co-existing pregnancy for female cases; (9) OCT findings on macular edema location i.e., center-involving DME (CIDME) or non-center-involving DME (NCIDME) and central macular thickness of both eyes, and (10) fundus fluorescein angiography findings on focal or diffuse leakage or macular ischemia, particularly in NCIDME cases.

The AI generated 25 hypothetical clinical patients and 50 clinical ocular case situations. They were then presented in a text format without any clinical images to a group of three experts (NR, NKY, SB) from various institutions to validate the clinical details and imaging findings in the case scenarios in order to make them appear as close to an actual clinical situation as possible. The experts’ recommendations were taken into account. The final version of the questionnaire identified 13 patients with identical ocular findings in both eyes. Thus, a total of 50 hypothetical case scenarios having 37 different ocular situations in 25 virtual patients was available for evaluation and analysis (Supplement 1). The final version of the clinical scenarios was then presented to a second group of five (VP, JC, RKR, PRK, DB) retina specialists/clinicians with at least five years of clinical experience in the field of retina, DR screening, and DME management. These retina specialists worked in a variety of settings, including government hospitals, independent private practices, tertiary eye care hospitals serving both free and paying patients, and tertiary eye care corporate hospitals serving only paying patients. Clinicians were asked to provide the single best treatment option for each eye in the case scenario, while keeping in mind that the patient was visiting the clinician for the first time for treatment and that the focus was on DME and ocular co-morbidity management. Any additional treatment for co-existing ocular co-morbidities like cataract, glaucoma, proliferative DR, etc., had to be mentioned separately as well. The maximum number of identical clinician responses in each category was used to determine the ‘majority DME management response’ and ‘majority ocular co-morbidity management response’ for the clinicians. Following that, the same set of clinical case scenarios was presented to three important AI platforms, which included ChatGPT 3.5v, ChatGPT 4.0v, and Bing AI. The text was fed into various AI platforms in such a way that the ophthalmologist appeared to be asking the AI for the best recommended treatment option based on the most recent available guidelines, with separate answers for each eye. After each case scenario, the AI received no feedback, and the case descriptions were entered sequentially without starting a new chat session. The formal responses for each case scenario were documented separately for DME management and co-existing ocular co-morbidity management based on the results generated by various AI platforms. For each specific case scenario, the ‘majority DME management response’ and ‘majority ocular co-morbidity management response’ were determined by identifying the most identical responses among the three distinct AI platforms in each category separately.

**Fig. 1:**
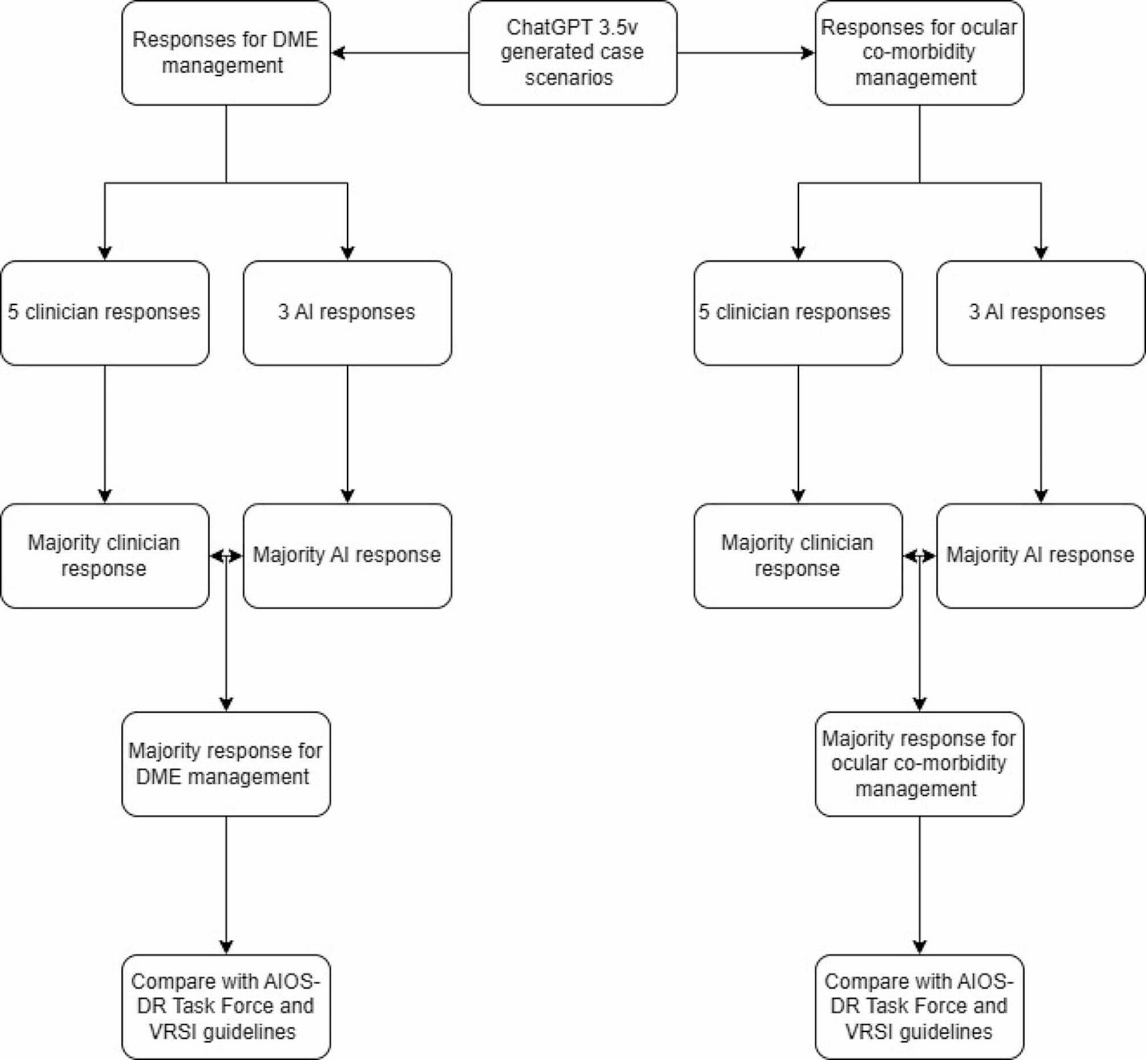
Flow chart depicting the methodology process

Based on clinician and AI platform responses, a consensus was reached on the most common response for each individual case scenario in order to determine the optimal treatment to be followed by a retina specialist in specific clinical situations. For each specific case scenario, the ‘majority’ DME management and ocular co-morbidity management responses were determined by identifying the response with the highest frequency among clinicians and AI platforms. A total of eight responses were considered, with five coming from clinicians and three from AI platforms **(**Fig. 1**)**.

The AIOS-DR Task Force and the VRSI collectively published a consensus paper on the management of DME. The paper suggested treatment guidelines for DME management in a variety of clinical situations involving ocular and systemic co-morbidities. We looked at the treatment practices recommended by our group of clinicians and different AI platforms for similar clinical situations described in that paper. For that, the clinical situations from our current case list were divided into 4 categories: (a) DME management in cases with NCIDME; (b) DME management in cases with treatment-naïve and previously treated CIDME; (c) DME management in cases with co-existing ocular co-morbidities and (d) DME management in cases with co-existing systemic co-morbidities. The purpose of this exercise was to see if recommendations suggested collectively by the clinicians and different AI platforms were consistent with the National guidelines for DME management.

Given the nature of the study, this research was exempted from further approvals by the institutional review board.

### Statistical analysis

The inter-rater reliability agreements among various clinicians, different AI platforms, and the ‘majority response’ for AI and clinician, separately for DME management and co-existing ocular co-morbidity management, were determined using Fleiss Kappa and Cohen’s Kappa (ĸ values) analysis. The calculations were performed on DATAtab: Online Statistics Calculator, developed by DATAtab E.U. in Graz, Austria. The calculator can be accessed at the following URL: https://datatab.net. The Kappa result is interpreted as follows: ĸ values ≤ 0 as indicating no agreement and 0.01–0.20 as none to slight, 0.21–0.40 as fair, 0.41– 0.60 as moderate, 0.61–0.80 as substantial, and 0.81–1.00 as almost perfect agreement [22]. 

## Results

### Inter-reliability agreement amongst and between clinicians and different AI platforms for management of DME

The Fleiss kappa test revealed that there was moderate agreement between the five clinicians, with ĸ = 0.60 (95% CI: 0.55–0.65). According to the Fleiss Kappa, there was a moderate agreement between ChatGPT 3.5, ChatGPT 4.0, and Bing AI, with ĸ = 0.58 (95% CI: 0.47–0.69). Cohen’s Kappa revealed a substantial agreement between ‘majority clinician response’ and ‘majority AI response’ for DME management, with ĸ = 0.69 (95% CI: 0.5–0.88). The Cohen’s kappa for the inter-rate reliability agreements between the individual AI platforms and the ‘majority clinician response’ was also calculated. ChatGPT 3.5v, ChatGPT 4.0v, and Bing AI had ĸ values of 0.5 (95% CI: 0.31–0.7), 0.61 (95% CI: 0.41–0.81), and 0.53 (95% CI: 0.34–0.72), respectively.

### Inter-reliability agreement amongst and between clinicians and different AI platforms for management of co-existing ocular co-morbidities

There was substantial agreement among the five clinicians for the management of co-existing ocular morbidities, with ĸ=0.80 (95% CI: 0.72–0.88). The Fleiss kappa revealed a fair agreement between ChatGPT 3.5, ChatGPT 4.0, and Bing AI, with ĸ= 0.36 (95% CI: 0.23–0.48). Cohen’s kappa analysis revealed a moderate agreement (ĸ=0.49) between the ‘majority clinician response’ and the ‘majority AI response’. Using Cohen’s kappa analysis, the inter-rater reliability agreements between the individual AI platforms and the ‘majority clinician response’ were calculated. ChatGPT 3.5v, ChatGPT 4.0v, and Bing AI had ĸ values of 0.6, 0.28, and 0.32, respectively **(**Tables 1 and 2**)**.

**Table 1:** ‘Majority’ responses by clinicians and AI platforms for individual case scenarios

Case	Eye	DME management		Ocular co-morbidity management	
		Majority clinician response	Majority AI response	Majority clinician response	Majority AI response
1	RE	ML	OBS	OBS	OBS
	LE	IVA	IVA	OBS	OBS
2	RE	ML	ML	PRP	PRP
	LE	ML	ML	PRP	PRP
3	RE	OBS	OBS	OBS	OBS
	LE	OBS	OBS	OBS	OBS
4	RE	OBS	OBS	OBS	OBS
	LE	OBS	IVA	PRP	OBS
5	RE	OBS	OBS	OBS	OBS
	LE	ML	ML	OBS	OBS
6	RE	OBS	OBS	OBS	OBS
	LE	IVA	IVA	OBS	OBS
7	RE	IVA	IVA	OBS	CAT
	LE	OBS	IVA	OBS	OBS
8	RE	IVS	IVA	PRP	OBS
	LE	OBS	OBS	PRP	OBS
9	RE	ML	ML	OBS	OBS
	LE	ML	ML	OBS	OBS
10	RE	IVS	IVA	OBS	OBS
	LE	IVS	IVA	OBS	OBS
11	RE	OBS	OBS	OBS	OBS
	LE	OBS	OBS	OBS	OBS
12	RE	IVA	IVA	OBS	OBS
	LE	OBS	OBS	OBS	OBS
13	RE	OBS	OBS	OBS	OBS
	LE	OBS	OBS	OBS	OBS
14	RE	OBS	OBS	OBS	OBS
	LE	OBS	OBS	OBS	OBS
15	RE	IVA	IVA	OBS	OBS
	LE	IVA	IVA	OBS	OBS
16	RE	IVS	ML	OBS	OBS
	LE	OBS	OBS	OBS	OBS
17	RE	OBS	IVA	PRP	OBS
	LE	OBS	OBS	PRP	PRP
18	RE	OBS	OBS	OBS	OBS
	LE	OBS	OBS	OBS	OBS
19	RE	IVA	IVA	OBS	OBS
	LE	IVA	IVA	OBS	OBS
20	RE	OBS	OBS	OBS	OBS
	LE	OBS	OBS	OBS	OBS
21	RE	IVA	IVA	OBS	OBS
	LE	OBS	OBS	OBS	OBS
22	RE	IVS	IVA	OBS	OBS
	LE	OBS	OBS	OBS	OBS
23	RE	IVA	IVA	OBS	OBS
	LE	IVA	IVA	PRP	CAT
24	RE	OBS	OBS	OBS	OBS
	LE	OBS	OBS	OBS	OBS
25	RE	IVA	IVA	OBS	OBS
	LE	IVA	IVA	OBS	OBS

Abbreviations: AI– artificial intelligence; DME– diabetic macular edema; RE– right eye; LE– left eye; OBS– observation; ML– macular laser; IVA– intravitreal antiVEGF; IVS– intravitreal steroid; PRP– pan retinal photocoagulation; CAT– cataract surgery

**Table 2:** Results of the Kappa analysis between clinicians and different AI platforms for DME management and ocular co-morbidity management for individual case situations:

Kappa analysis between	DME management (ĸ value)	Ocular co-morbidity management (ĸ value)
1) Clinicians	0.60	0.80
2) AI platforms	0.58	0.36
3) Majority Clinician and majority AI response	0.69	0.49
4) Majority Clinician response and ChatGPT3.5v	0.50	0.60
5) Majority Clinician response and ChatGPT 4.0v	0.61	0.28
6) Majority Clinician response and Bing AI	0.53	0.32

Abbreviations: AI– artificial intelligence; DME– diabetic macular edema; ĸ– kappa

Further analysis of case scenarios was performed to determine whether the recommendations for DME management generated jointly by the clinician and AI were consistent with the National guidelines:

1. Management of NCIDME:

In our study, we discovered eight (16%) eyes with NCIDME. These eyes all had 20/30 or better VA. In our study, the most common response for this specific situation was observation (*n* = 7, 88%) for DME management.

2. Management of CIDME:

This study identified 22 (44%) eyes with CIDME. Half of the eyes (*n* = 11, 50%) had untreated CIDME, while the others had already been treated. Four (36%) of the eleven eyes had vision better than 20/30, four (36%) had visual acuities between 20/30 and 20/40, and three (28%) had vision worse than 20/50. Treatment with intravitreal injections was advised in three (75%) of the four eyes with VA ≥ 20/30. Intravitreal injections remained the most preferred treatment option for DME management in eyes with visual acuities ranging from 20/30 to 20/40 (3 out of 4 cases, 75%). In eyes with reduced VA, i.e., 20/50, intravitreal pharmacotherapeutic agents were the only treatment option. Only one (12%) of the eight eyes with good VA, i.e., vision acuity ≥ 20/40, had no visual symptoms. Patients reported visual symptoms in the remaining 7 (88%) cases. Treatment was considered in all eight eyes with good VA, regardless of visual complaints, according to the joint recommendations in our study.

The remaining 11 (50%) eyes with CIDME had previously been treated for DME. Six (55%) eyes had very good VA ranging from 20/20 to 20/30, three (27%) eyes had good VA ranging from 20/30 to 20/40, and two eyes (18%) had reduced VA, i.e., worse than 20/50. Two-thirds (4 eyes, 67%) of the eyes with very good VA, i.e., better than 20/30, were treated with intravitreal injections of steroids or anti vascular endothelial growth factors (VEGF) agents. Only intravitreal pharmacotherapeutic agents were used to treat eyes with VA ≤ 20/40.

3. Management of CIDME with co-existing ocular co-morbidities:

Six (27%) of the 22 CIDME eyes also had proliferative DR. In such cases, DME was treated with intravitreal antiVEGF injections in three (50%) eyes, intravitreal steroids in one (17%) eye, and macular laser therapy in two (33%) eyes. Macular laser was preferred in two cases where the patient was pregnant at the time. The current study included six (27%) eyes with pseudophakia and CIDME. Treatment for DME was primarily considered with intravitreal steroid agents in four (67%) eyes and intravitreal antiVEGF agents in two (33%) eyes. The remaining 16 (73%) eyes were all phakic. Significant cataract was found in ten (63%) of the sixteen eyes. The rest of the lenses were clear. Treatment with an intravitreal antiVEGF agent was the only preferred treatment of choice in eyes with significant cataract for the management of DME. Cataract surgery was not considered in any of the ten eyes at the same time as intravitreal injection. None of the eyes with CIDME in the current case list had co-existing glaucoma. In the current case list, there was only one eye (5%) with CIDME that had previously undergone pars plana vitrectomy surgery. There were 2 (9%) eyes in the current study who had CIDME and co-existing peripheral lattice degeneration. Prophylactic barrage laser was not advised in both these cases prior to treatment with intravitreal injections.

4. Management of CIDME with co-existing systemic abnormalities:

There were 15 (68%) eyes with CIDME in the current list of case scenarios that had poor diabetes control, i.e., HbA1c > 6.5%. In none of the clinical situations was DME management withheld in order to achieve metabolic control of diabetes. There were eleven (50%) eyes that had CIDME as well as renal disease and anemia. In such eyes, the most preferred treatment for CIDME was intravitreal antiVEGF agents in 8 eyes (73%) and intravitreal steroids in 3 (27%) eyes. In no case was DME treatment postponed to allow for renal status control. There were 18 (82%) eyes of CIDME with co-existing hypertension on the current list of cases. According to the clinicians’ and AI’s joint recommendations, treatment for CIDME with hypertension in the form of macular laser or intravitreal injections was considered immediately. There were five (23%) eyes with CIDME and a pregnancy. In these eyes, DME was treated with either a macular laser in four (80%) of them or intravitreal steroids in one (20%). In eyes with CIDME and concurrent pregnancy, an intravitreal antiVEGF agent was not considered the best treatment option. Thirteen (43%) of the current cases had DME and a history of cardiovascular disease. Eleven (50%) of these eyes had CIDME, and three (27%) of them had a history of cardiovascular disease within the previous three months. In patients with cardiovascular disease, regardless of its recent history, intravitreal antiVEGF injections were the preferred treatment option.

## Discussion

In summary, this one-of-a-kind study involved noting treatment suggestions for DME and ocular co-morbidity management separately to a set of ocular case scenarios generated by the AI, comparing the responses provided by clinicians and different AI platforms to different clinical situations, and finally match the collective responses provided by the different AI platforms and clinicians to different clinical situations with the previously published recommendations by the AIOS-DR Task Force and the VRSI.

The prevalence of DME, as well as the availability of trained medical personnel, retinal imaging tools, and management options, varies by geographic region [4, 23, 24]. Furthermore, treatment practices differ significantly within a defined region based on a variety of factors such as the type of patient (urban versus rural), the patient’s economic status and countries resource settings, and the availability of treatment options such as intravitreal injections or lasers. As a result, different parts of the world establish their own treatment guidelines for DME management [8–13]. The International Council of Ophthalmology Guidelines for Diabetic Eye Care 2017 summarised and provided a comprehensive guide for DR screening, referral and follow-up schedules, and appropriate management of vision-threatening DR, including DME and proliferative DR, for countries with high, low, or intermediate resource levels [25]. We found varying levels of agreement among clinicians in this study for the management of DME as well as the management of ocular co-morbidities. The clinicians who participated in this study provided responses from different regions of the country, treating different groups of patients with varying social and economic backgrounds, which explains the varying levels of agreement among the clinicians.

The current role of AI in DME management is primarily limited to identifying and classifying DME using color fundus photographs and/or optical coherence tomography images, as well as predicting the response to antiVEGF therapy using machine learning or deep learning models [14, 15, 26]. Several chatbots developed using LLM-based generative AI applications have shown promising results in generalizing to previously unseen tasks, such as medical question-answering requiring scientific expert knowledge [27–29]. LLM understands the medical context, recalls and interprets relevant medical information, and produces a response in a text-based format in order to formulate an answer. Despite mixed results in ophthalmology, LLM appears to have potential for use in eye health care applications. LLM-based generative AI with ChatGPT3.5v and ChatGPT 4.0v has been used in retina for a variety of indications, including ICD for various case encounters [30, 31]. The use of LLM-based generative AI for DME management recommendations in the presence of other ocular co-morbidities has yet to be investigated. Furthermore, different chatbot applications react differently to the same situations [32]. Even in this study, the different AI platforms demonstrated varying levels of agreement for the same clinical case scenario. There was moderate agreement among the different AI platforms for the management of DME and fair agreement for the management of co-existing ocular morbidities. To address this issue, the ‘majority AI response’ was chosen as the preferred method for managing the DME using AI. To improve both the precision and speed of responses, the AI platform must have real-time access to the internet and receive the most up-to-date information. In this study, we discovered that ChatGPT 4.0v performed better and had closer agreements with clinician responses than the other two AI platforms for DME management, while ChatGPT3.5v performed better for co-existing ocular comorbidities management.

A complete ophthalmic examination, as well as a thorough ocular and systemic history, are required for DME management. Most globally accepted treatment guidelines for DME management available in the literature, including protocols developed by the Diabetic Retinopathy Clinical Research Network (DRCR.net), use limited criteria for guiding DME treatment, such as metabolic control status, VA, treatment-naive status, and the involvement of a 1-mm central subfield region on OCT by retinal thickening [8–13, 33, 34]. The AIOS-DR Task Force and the VRSI recently published consensus guidelines in 2021 that looked at some additional criteria such as the presence of co-existing ocular and systemic co-morbidities in addition to the above-mentioned criteria when planning DME management [13]. As a result, we compared the recommendations made by the clinicians and AI in this study for various clinical case scenarios to the consensus recommendations made by the AIOS-DR Task Force and the VRSI.

According to the current study recommendations, most eyes with NCIDME and good VA (i.e., 20/30) were only observed or rarely treated with topical non-steroidal anti-inflammatory drugs. According to the recommendations of the AIOS-DR Task Force and the VRSI, eyes with good VA should be observed and followed up on at monthly intervals to look for conversion to CIDME or deterioration in VA [13]. According to protocol R of the DRCR.net, topical non-steroidal anti-inflammatory drugs have no noticeable effect in eyes with NCIDME and good VA [35]. 

Intravitreal antiVEGF injections are the first line of treatment for naive CIDME [36, 37]. VA is the most important criterion for deciding to initiate treatment, selecting the right intravitreal pharmacotherapeutic antiVEGF agent for its management, and for prognosis purposes, according to most well-established DME treatment guidelines [8–13, 33, 34]. According to DRCR.net protocol V, eyes with treatment-naive CIDME and very good VA, i.e., ≥ 20/30, can be observed and followed up on a monthly basis instead of being treated with intravitreal antiVEGF injections [38]. In practice, however, treatment of naive CIDME eyes with very good VA is typically initiated with intravitreal antiVEGF injections only if the patient complains of visual symptoms. Intravitreal antiVEGF agents are typically used to treat naive CIDME eyes with VA below 20/30. The presenting VA also influences the choice of antiVEGF agents; for example, in eyes with acuity ≤ 20/50, intravitreal aflibercept injection is the preferred treatment option [39]. Regardless of presenting VA or patient visual symptoms, intravitreal antiVEGF injection was the only preferred treatment option in the current study recommendations for naive CIDME eyes. In the current study, intravitreal injections, either with antiVEGF agents or steroids, remained the mainstay of DME management for previously treated CIDME eyes. Intravitreal steroids are typically reserved for eyes that have persistent DME or do not respond to monthly antiVEGF injections [40, 41]. 

Proliferative DR and DME are both distinct patterns of retinal microvascular features indicative of small-vessel disease. Treatment-naive proliferative DR should be treated with pan-retinal photocoagulation, according to the AIOS-DR Task Force and the VRSI guidelines [13]. The presence or absence of vision-threatening traction determines the management regime of CIDME treatment in proliferative DR eyes. Traction that threatens or involves the fovea is an indication for vitrectomy surgery. Intravitreal injections, however, remain the mainstay of DME treatment in the presence of extramacular traction [42]. Intravitreal steroids are usually preferred in proliferative DR and DME eyes with extensive extramacular proliferations because intravitreal antiVEGF injections can worsen traction due to the crunch phenomenon [43]. The current study suggests that eyes with DME and proliferative DR without advanced fibrovascular proliferation be treated with intravitreal antiVEGF injections, whereas intravitreal steroid injections are preferred in DME eyes with proliferative DR and extensive fibrovascular proliferation.

The current study included a high proportion of cases with clear lenses, cataractous lenses, and pseudophakia, all of which co-existed with CIDME. In the absence of a visually disabling cataract, the AIOS-DR Task Force and the VRSI recommend that DME be stabilized first with intravitreal injections. However, in the presence of clinically significant cataracts with a poor view of the fundus, and preexisting DME, surgery along with intravitreal antiVEGF or steroid injections can be planned. In some cases, treatment can be scheduled two weeks after surgery and the subsequent protocol be followed [13]. The current study considered treating DME with intravitreal antiVEGF injections prior to cataract surgery. Vision in the study’s case scenarios ranged from 20/25 to 20/80, indicating moderate vision loss and visually insignificant cataract. This could be the reason why these eyes were treated for DME prior to cataract surgery rather than concurrently with cataract surgery. In the current study, intravitreal steroids were the preferred choice for DME management in pseudophakic eyes in two-thirds of the cases. According to national guidelines, the first step is to determine whether the macular edema in pseudophakic eyes is caused by DME or by Irvine-Gass syndrome. In the presence of DME without the presence of Irvine-Gass syndrome, treatment with antiVEGF injections can be initiated for CIDME, whereas topical or sub tenon’s steroids are recommended as first-line therapy for pseudophakic edema. Topical nonsteroidal anti-inflammatory drugs should be used first, followed by antiVEGF, in the presence of both DME and Irvine-Gass syndrome. It is reasonable to switch to steroids in eyes that have not responded to previous antiVEGF injections (after 3–6 injections) [13]. Many international experts around the world believe that intravitreal steroids injection with dexamethasone implant is a viable alternative first-line treatment option, particularly in pseudophakic eyes [44]. 

National guidelines recommend treating DME in eyes with established glaucoma, ocular hypertension, or steroid responders with either macular laser or antiVEGF injections. In these patients, steroids should be avoided [13]. There were no cases of CIDME and co-existing glaucoma in the current study’s case scenarios. As a result, comparisons with the National guidelines were not possible in this study. According to experts affiliated with the National guidelines, careful examination of the retinal periphery for identifying lesions that may predispose to retinal detachment, as well as prophylactic barrage laser treatment of those lesions, is recommended. In addition, the time between laser prophylaxis and antiVEGF injections should ideally be three weeks [13]. However, the current study’s joint experts (AI and clinicians) did not recommend prophylactic laser barrage to lattice degenerations prior to beginning DME treatment with intravitreal injections.

DME treatment in a vitrectomised eye is difficult. There is limited data on the preferred agent for treatment in these eyes. Furthermore, as the environment of the vitreous cavity changes, the pharmacokinetic parameters of antiVEGF may be affected. A recent study comparing the efficacy of ranibizumab injections for the treatment of DME in eyes with and without previous vitrectomy over a two-year period found similar results [45]. In a similar study, Koyanagi et al. found no significant differences in the mean changes in VA and central macular thickness between the two groups after 6 months [46]. However, some studies show that intravitreal antiVEGF injections have a lower efficacy in vitrectomised eyes [47]. Intravitreal dexamethasone implant has also been shown to be effective in both vitrectomised and non-vitrectomised eyes [48, 49]. Thus, current evidence suggests that both antiVEGF and steroids have a role in the treatment of DME in vitrectomised eyes.

Poor glycemic control is a risk factor for the progression of DR and DME. Strict glycemic control is beneficial at any stage of diabetes. Poor or fluctuating glycemic control can affect adherence to monthly intravitreal injections. However, in order to achieve good glycemic control, DME management should not be delayed. In the current study, DME management was not postponed in cases of poor diabetes control.

Little is known about the treatment of DME during pregnancy [50]. According to experts, watchful waiting may be used in cases of mild to moderate DME because the outcomes are similar in patients who receive prompt versus delayed DME treatment [51]. Because of potential adverse effects on developing embryos or foetuses, intravitreal antiVEGF injections are not recommended during pregnancy. Women should therefore wait at least three months after their last intravitreal injection before conceiving. Ranibizumab is usually preferred over other antiVEGF agents because of its shorter half-life and faster plasma clearance [52]. When the disease permits it, focal laser or intravitreal steroids are the preferred treatment options for DME in pregnancy [50, 51]. 

The current study’s clinicians and AI jointly proposed the use of intravitreal antiVEGF or steroid injection in eyes with CIDME and deranged renal status. Diabetic nephropathy is often associated with other systemic co-morbidities that affect DME. Secondary hypertension, anemia, patients on or after dialysis, and renal transplantation, for example, may all have an impact on the presence of DME and its management [53–55]. VEGF inhibitors may have nephrotoxic effects, according to emerging evidence [56]. However, a recent study published by Ku et al. found no correlation between the use of intravitreal antiVEGF injections and a decrease in estimated glomerular filtration rate [57]. Several DME treatment guidelines recommend identifying the cause of hypertension and controlling it before beginning treatment with intravitreal antiVEGF injections [8, 9, 12, 13]. AntiVEGF injections are effective in eyes with DME and well-controlled hypertension [58]. According to studies, patients with DME or proliferative DR are more likely to have incident or fatal cardiovascular disease than those who do not have DME or PDR [59]. If a patient has had a stroke or myocardial infarction within the previous 3 months, the AIOS-DR Task Force and the VRSI recommend that antiVEGF treatment be avoided; instead, PRP or steroid treatments should be considered in these patients [13]. However, a recent systematic review and meta-analysis published by Ngo Ntjam et al. examined the incidence of major cardiovascular adverse events in patients receiving intravitreal administration of anti-vascular endothelial growth factor drugs. They confirmed that intravitreal antiVEGF injections did not cause serious cardiovascular events [60]. Even in this study, intravitreal antiVEGF injections were the preferred treatment option in DME patients with a history of cardiovascular disease. Table 3 summarizes study’s joint recommendations for managing DME and provides comparisons with the national recommendations for DME management.

**Table 3:** Comparisons between the current study recommendations and national guidelines for different clinical situations for diabetic macular edema management

Category	Clinical situation	Current study recommendations	AIOS-DR Task Force and VRSI recommendations
DME status	No DME (*n* = 20)	Observation	Observation
	NCIDME (*n* = 8)	Observation	Observation
	Treatment naïve CIDME (*n* = 11)	IVA	Observation for eyes with very good VA IVA for VA < 20/30
	Previously treated CIDME (*n* = 11)	IVA or IVS	IVA
CIDME with co-existing ocular co-morbidity	CIDME with proliferative DR (*n* = 6)	IVA	IVA
	CIDME with pseudophakia (*n* = 6)	IVS	IVA or IVS or topical steroids
	CIDME with cataract (*n* = 10)	IVA	IVA with or without cataract surgery
	CIDME with glaucoma (*n* = 0)	Cannot be commented upon	Avoid IVS
	CIDME with LD (*n* = 2)	IVA or IVS without prophylactic barrage to the LD	Prophylactic barrage to the LD first followed by IVA or IVS after 3 weeks
	CIDME in vitrectomised eye (*n* = 1)	IVS	IVA or IVS
CIDME with co-existing systemic co-morbidity	CIDME with uncontrolled diabetes (*n* = 15)	IVA or IVS irrespective good metabolic control	IVA or IVS with good metabolic control
	CIDME with renal disease and anemia (*n* = 11)	IVA	Controversial role of IVA
	CIDME with hypertension (*n* = 18)	IVA or IVS	IVA with good control of blood pressure
	CIDME with pregnancy (*n* = 5)	Macular laser or IVS	Observation or focal laser or IVS. Avoid IVA
	CIDME with history of CVD within 3 months (*n* = 3)	IVA	Avoid IVA

Abbreviations: DME– diabetic macular edema; DR– diabetic retinopathy; AIOS– All-India Ophthalmology Society; VRSI– Vitreo-Retina Society of India; CIDME– center-involving diabetic macular edema; NCIDME– non-center-involving diabetic macular edema; IVA– intravitreal anti vascular endothelial growth factor; VA– visual acuity; IVS– intravitreal steroids; LD– lattice degeneration; CVD– cardiovascular disease

There are a few weaknesses with this study. Regarding the clinical case scenarios generated by the AI, the number of case scenarios provided could have been greater, taking into account all possible permutations and combinations involving the DME status, as well as the ocular and systemic co-morbidity status. The clinical case scenarios could have been presented to a larger group of clinicians on a national and international scale, as well as to multiple AI applications. The term ‘majority’ clinician and AI responses used in this study may mislead readers and be misinterpreted as the best response from the entire fraternity of retina specialists and different AI platforms. In the current study, the AI and clinician responses were only compared to the National guidelines proposed by a group of experts affiliated with the AIOS-DR Task Force and the VRSI. The current study’s recommendations could have been compared to international DME management guidelines, making the study’s findings more globally acceptable. Nonetheless, the study has the advantage of adding a completely new dimension to the formulation of DME treatment guidelines by incorporating the role of LLM-based generative AI. The study also emphasizes the importance of considering the patient’s eye and body as a whole when planning DME management. It is possible to make these recommendations globally acceptable by addressing the aforementioned flaws in future DME and AI-related studies. Furthermore, in the future, these globally acceptable DME management recommendations could be integrated with the hospital’s electronic medical record system, alerting ophthalmologists to the best possible treatment option after considering the various factors that may influence DME developments and management. The AI has the capability to analyse and assess its recommendations based on the specific approaches used by different clinicians for managing DME by setting up prompts on the electronic medical record. In the future, AI will incorporate self-learning algorithms tailored to each clinician’s practices, allowing the algorithm to learn from a clinician’s recommendations for a particular patient. This adaptive learning process will help enhance the algorithm’s performance.

In conclusion, this study highlights the significance of AI in aiding experts in revising the existing treatment guidelines for managing DME. An optimal approach for the future would involve merging these treatment guidelines with the hospital’s electronic medical record software, enabling clinicians to promptly select the most effective treatment option for managing DME.

### Electronic supplementary material

Below is the link to the electronic supplementary material.

Supplementary Material 1

## Data Availability

The datasets used and/or analysed during the current study are available from the corresponding author on reasonable request.

## Declarations

### Animal research

This article does not contain any studies with animals performed by any of the authors.

## Abbreviations

DME: diabetic macular edema
DR: diabetic retinopathy
AI: artificial intelligence
LLM: large language model
AIOS: All-India Ophthalmological Society
VRSI: Vitreo-Retina Society of India
OCT: optical coherence tomography
VA: visual acuity
CIDME: center-involving diabetic macular edema
NCIDME: non-center involving diabetic macular edema
VEGF: vascular endothelial growth factor
DRCR.net: Diabetic Retinopathy Clinical Research Network
