# Supplementary material for: Recommendations for diabetic macular edema management by retina specialists and large language model-based artificial intelligence platforms
Source: Int J Retina Vitreous. 2024 Feb 28;10:22. doi: 10.1186/s40942-024-00544-6 (PMC10900631; doi:10.1186/s40942-024-00544-6)
Supplement: Supplementary file 1 — Supplementary Material 1 [file 40942_2024_544_MOESM1_ESM.pdf]

Case Scenario 1:

1) **Patient Demographics:**
   - Age: 55 years
   - Gender: Male

2) **Diabetes Details:**
   - Type: Type 2 diabetes
   - Duration: 15 years
   - Control: HbA1c 7.2% on oral hypoglycemic agents

3) **Systemic Co-morbidities:**
   - Renal Disease: Stage 2 chronic kidney disease
   - Hypertension: Well-controlled with ACE inhibitor
   - Anemia: Mild, managed with iron supplements
   - Cardiovascular Disease: History of myocardial infarction 3 months back, on aspirin and statin
   - Lipid Profile: Elevated LDL, managed with statin

4) **Pregnancy Status:**
   - Not applicable (male patient)

5) **Visual Symptoms:**
   - Recent onset of blurry vision and difficulty reading in both eyes

6) **Visual Acuity:**
   - Right Eye: 20/30
   - Left Eye: 20/40

7) **Lens Status:**
   - Early cataract in both eyes

8) **Glaucoma Status:**
   - No pre-existing glaucoma

9) **Previous Treatment:**
   - No history of laser, anti-VEGF, or vitrectomy

10) **Fundus Findings:**
   - Non-Proliferative Diabetic Retinopathy (NPDR) in both eyes with microaneurysms and hard exudates

11) **OCT Findings:**
   - Center-involving macular edema in the left eye with a central macular thickness of 400 microns; right eye has non-center involving edema with a thickness of 320 microns

12) **FFA Findings:**
   - Diffuse leakage in the left eye with minimal enlargement of the foveal avascular zone; right eye shows focal leakage in the macula

Clinician's treatment plan:

Eye	Clinician's single best response for DME management	Clinician's single best treatment response for ocular co-morbidity management:
RE		
LE		

Case Scenario 2:

1) **Patient Demographics:**
- Age: 32 years
- Gender: Female

2) **Diabetes Details:**
- Type: Type 1 diabetes
- Duration: 20 years
- Control: HbA1c 8.5% on insulin therapy

3) **Systemic Co-morbidities:**
- Renal Disease: Normal renal function
- Hypertension: Controlled with beta-blocker
- Anemia: No anemia
- Cardiovascular Disease: No history
- Lipid Profile: Within normal limits

4) **Pregnancy Status:**
- Co-existing pregnancy in the second trimester

5) **Visual Symptoms:**
- Blurred vision and distorted images

6) **Visual Acuity:**
- Right Eye: 20/25
- Left Eye: 20/30

7) **Lens Status:**
- Clear lens in both eyes

8) **Glaucoma Status:**
- No pre-existing glaucoma

9) **Previous Treatment:**
- Previous focal laser therapy for macular edema in both eyes during the pre-pregnancy period more than 6 months back

10) **Fundus Findings:**
- Proliferative Diabetic Retinopathy (PDR) in both eyes with new vessels and fibrous proliferation

11) **OCT Findings:**
- Center-involving macular edema in both eyes with a central macular thickness of 380 microns

12) **FFA Findings:**
- Focal leakage in the macula of both eyes

Clinician’s treatment plan:

Eye	Clinician’s single best response for DME management	Clinician’s single best treatment response for ocular co-morbidity management:
RE		
LE		

Case Scenario 3:

1) **Patient Demographics:**
   - Age: 68 years
   - Gender: Female

2) **Diabetes Details:**
   - Type: Type 2 diabetes
   - Duration: 25 years
   - Control: HbA1c 8.9% on insulin therapy

3) **Systemic Co-morbidities:**
   - Renal Disease: End-stage renal disease on hemodialysis
   - Hypertension: Poorly controlled despite multiple antihypertensive medications
   - Anemia: Severe, Hb = 6.9gm/dl
   - Cardiovascular Disease: History of stroke, on anticoagulation
   - Lipid Profile: Elevated triglycerides and low HDL, difficult to manage

4) **Pregnancy Status:**
   - Not applicable (postmenopausal)

5) **Visual Symptoms:**
   - No recent onset visual symptoms

6) **Visual Acuity:**
   - Right Eye: 20/25
   - Left Eye: 20/30

7) **Lens Status:**
   - Pseudophakia in both eyes

8) **Glaucoma Status:**
   - History of narrow-angle glaucoma, well-controlled with laser peripheral iridotomy

9) **Previous Treatment:**
   - Past history of pan retinal laser photocoagulation done 6 months prior for proliferative diabetic retinopathy (PDR) in both eyes

10) **Fundus Findings:**
   - Stable PDR with fibrous proliferation, no active neovascularization

11) **OCT Findings:**
   - No macular edema in either eye

12) **FFA Findings:**
   - No evidence of macular ischemia, no leakage on fluorescein angiography

Clinician’s treatment plan:

Eye	Clinician’s single best response for DME management	Clinician’s single best treatment response for ocular co-morbidity management:
RE		
LE		

Case Scenario 4:

1) **Patient Demographics:**
   - Age: 45 years
   - Gender: Male

2) **Diabetes Details:**
   - Type: Type 1 diabetes
   - Duration: 30 years
   - Control: HbA1c 7.8% on insulin pump therapy

3) **Systemic Co-morbidities:**
   - Renal Disease: Microalbuminuria
   - Hypertension: Well-controlled with ACE inhibitor
   - Anemia: Mild, no intervention required
   - Cardiovascular Disease: No history
   - Lipid Profile: Elevated LDL, managed with statin

4) **Pregnancy Status:**
   - Not applicable (male patient)

5) **Visual Symptoms:**
   - Sudden onset floaters and flashes of light

6) **Visual Acuity:**
   - Right Eye: 20/20
   - Left Eye: 20/25

7) **Lens Status:**
   - Clear lens

8) **Glaucoma Status:**
   - No pre-existing glaucoma

9) **Previous Treatment:**
   - No previous ocular interventions

10) **Fundus Findings:**
   - Acute onset minimal vitreous hemorrhage in the left eye with evidence of active neovascularization

11) **OCT Findings:**
   - No evidence of macular edema in right eye and presence of non-CI DME in left eye

12) **FFA Findings: **
   - Leakage in the left eye, indicating active neovascularization

Clinician's treatment plan:

Eye	Clinician's single best response for DME management	Clinician's single best treatment response for ocular co-morbidity management:
RE		
LE		

Case Scenario 5:

1) **Patient Demographics:**
   - Age: 60 years
   - Gender: Female

2) **Diabetes Details:**
   - Type: Type 2 diabetes
   - Duration: 18 years
   - Control: HbA1c 7.0% on oral hypoglycemic agents

3) **Systemic Co-morbidities:**
   - Renal Disease: Mild proteinuria
   - Hypertension: Well-controlled with angiotensin receptor blocker
   - Anemia: Moderate, managed with iron and erythropoietin
   - Cardiovascular Disease: History of coronary artery bypass surgery, 2 years back
   - Lipid Profile: Within normal limits

4) **Pregnancy Status:**
   - Not applicable (postmenopausal)

5) **Visual Symptoms:**
   - Gradual loss of peripheral vision

6) **Visual Acuity:**
   - Right Eye: 20/25
   - Left Eye: 20/30

7) **Lens Status:**
   - Early cataract in both eyes

8) **Glaucoma Status:**
   - Diagnosed with primary open-angle glaucoma, well-controlled with prostaglandin analogues

9) **Previous Treatment:**
   - History of focal laser for macular edema in the right eye

10) **Fundus Findings:**
    - Moderate non-proliferative diabetic retinopathy (NPDR) in both eyes with cotton-wool spots

11) **OCT Findings:**
    - Non-center involving macular edema in the left eye with a central macular thickness of 320 microns; right eye has no macular edema

12) **FFA Findings:**
    - Focal leakage in the left eye

Clinician’s treatment plan:

Eye	Clinician’s single best response for DME management	Clinician’s single best treatment response for ocular co-morbidity management:
RE		
LE		

Case Scenario 6:

1) **Patient Demographics:**
   - Age: 40 years
   - Gender: Male

2) **Diabetes Details:**
   - Type: Type 1 diabetes
   - Duration: 10 years
   - Control: HbA1c 7.5% on multiple daily injections

3) **Systemic Co-morbidities:**
   - Renal Disease: Normal renal function
   - Hypertension: Controlled with ACE inhibitor
   - Anemia: No anemia
   - Cardiovascular Disease: No history
   - Lipid Profile: Within normal limits

4) **Pregnancy Status:**
   - Not applicable (male patient)

5) **Visual Symptoms:**
   - No recent onset visual symptoms

6) **Visual Acuity:**
   - Right Eye: 20/20
   - Left Eye: 20/25

7) **Lens Status:**
   - No cataract, pseudophakia, or aphakia

8) **Glaucoma Status:**
   - No pre-existing glaucoma

9) **Previous Treatment:**
   - No previous ocular interventions

10) **Fundus Findings:**
   - No diabetic retinopathy, but evidence of epiretinal membrane in the left eye

11) **OCT Findings:**
   - Center-involving macular edema in the left eye with a central macular thickness of 420 microns; right eye has no macular edema

12) **FFA Findings:**
   - Macular leakage in the left eye

Clinician's treatment plan:

Eye	Clinician's single best response for DME management	Clinician's single best treatment response for ocular co-morbidity management:
RE		
LE		

Case Scenario 7:

1) **Patient Demographics:**
   - Age: 55 years
   - Gender: Female

2) **Diabetes Details:**
   - Type: Type 2 diabetes
   - Duration: 12 years
   - Control: HbA1c 6.5% on lifestyle modification

3) **Systemic Co-morbidities:**
   - Renal Disease: No evidence of renal dysfunction
   - Hypertension: Well-controlled with diuretics
   - Anemia: Mild, managed with oral iron supplementation
   - Cardiovascular Disease: No history
   - Lipid Profile: Within normal limits

4) **Pregnancy Status:**
   - Not applicable (postmenopausal)

5) **Visual Symptoms:**
   - Sudden onset of floaters and dark spots in vision

6) **Visual Acuity:**
   - Right Eye: 20/50
   - Left Eye: 20/30

7) **Lens Status:**
   - Early cataract in the right eye; pseudophakia in the left eye

8) **Glaucoma Status:**
   - No pre-existing glaucoma

9) **Previous Treatment:**
   - No h/o of any previous treatment

10) **Fundus Findings:**
   - Severe non-proliferative DR in both eyes with RE CSME and RE has inferior peripheral lattice degeneration

11) **OCT Findings:**
   - Center-involving DME in right eye and left eye showing non-center involving DME

12) **FFA Findings:**
   - Both eyes show diffuse macular leakage

Clinician's treatment plan:

Eye	Clinician's single best response for DME management	Clinician's single best treatment response for ocular co-morbidity management:
RE		
LE		

Case Scenario 8:

1) **Patient Demographics:**
   - Age: 70 years
   - Gender: Male

2) **Diabetes Details:**
   - Type: Type 2 diabetes
   - Duration: 30 years
   - Control: HbA1c 8.2% on insulin therapy

3) **Systemic Co-morbidities:**
   - Renal Disease: Moderate chronic kidney disease
   - Hypertension: Poorly controlled despite multiple antihypertensive medications
   - Anemia: Severe, requiring erythropoietin therapy
   - Cardiovascular Disease: History of heart failure, 3 years back on medications
   - Lipid Profile: Elevated triglycerides and low HDL, difficult to manage

4) **Pregnancy Status:**
   - Not applicable (male patient)

5) **Visual Symptoms:**
   - Gradual loss of central vision

6) **Visual Acuity:**
   - Right Eye: 20/200
   - Left Eye: 20/30

7) **Lens Status:**
   - Pseudophakia in both eyes

8) **Glaucoma Status:**
   - No pre-existing glaucoma

9) **Previous Treatment:**
   - Previous intravitreal anti-VEGF injections for diabetic macular edema in both eyes

10) **Fundus Findings:**
   - Bilateral advanced proliferative diabetic retinopathy (PDR)

11) **OCT Findings:**
   - Severe center-involving macular edema in the right eye with a central macular thickness of 600 microns; left eye has no macular edema

12) **FFA Findings:**
   - Right eye shows diffuse macular leakage and left eye has no macular leakage

Clinician's treatment plan:

Eye	Clinician's single best response for DME management	Clinician's single best treatment response for ocular co-morbidity management:
RE		
LE		

Case Scenario 9:

1) **Patient Demographics:**
   - Age: 48 years
   - Gender: Female

2) **Diabetes Details:**
   - Type: Type 1 diabetes
   - Duration: 22 years
   - Control: HbA1c 7.0% on insulin pump therapy

3) **Systemic Co-morbidities:**
   - Renal Disease: Normal renal function
   - Hypertension: Well-controlled with angiotensin receptor blocker
   - Anemia: No anemia
   - Cardiovascular Disease: No history
   - Lipid Profile: Within normal limits

4) **Pregnancy Status:**
   - Currently in the first trimester of pregnancy

5) **Visual Symptoms:**
   - Blurred vision and photophobia

6) **Visual Acuity:**
   - Right Eye: 20/40
   - Left Eye: 20/30

7) **Lens Status:**
   - No cataract, pseudophakia, or aphakia

8) **Glaucoma Status:**
   - No pre-existing glaucoma

9) **Previous Treatment:**
   - No previous ocular interventions

10) **Fundus Findings:**
   - Bilateral non-proliferative diabetic retinopathy (NPDR) with macular edema

11) **OCT Findings:**
   - Center-involving macular edema in both eyes with a central macular thickness of 350 microns

12) **FFA Findings:**
   - Focal leakage in the macula of both eyes

Clinician's treatment plan:

Eye	Clinician's single best response for DME management	Clinician's single best treatment response for ocular co-morbidity management:
RE		
LE		

Case Scenario 10:

1) **Patient Demographics:**
   - Age: 62 years
   - Gender: Male

2) **Diabetes Details:**
   - Type: Type 2 diabetes
   - Duration: 20 years
   - Control: HbA1c 7.5% on oral hypoglycemic agents

3) **Systemic Co-morbidities:**
   - Renal Disease: Stage 3 chronic kidney disease
   - Hypertension: Controlled with ACE inhibitor
   - Anemia: Moderate, managed with erythropoietin therapy
   - Cardiovascular Disease: History of coronary artery disease, on antiplatelet therapy
   - Lipid Profile: Elevated LDL, managed with statin

4) **Pregnancy Status:**
   - Not applicable (male patient)

5) **Visual Symptoms:**
   - Fluctuating vision and difficulty in low-light conditions

6) **Visual Acuity:**
   - Right Eye: 20/30
   - Left Eye: 20/40

7) **Lens Status:**
   - Pseudophakia in both eyes

8) **Glaucoma Status:**
   - No pre-existing glaucoma

9) **Previous Treatment:**
   - Previous intravitreal steroid injections for refractory diabetic macular edema in both eyes

10) **Fundus Findings:**
   - Bilateral severe non-proliferative diabetic retinopathy (NPDR) with macular edema

11) **OCT Findings:**
   - Center-involving macular edema in both eyes with a central macular thickness of 450 microns

12) **FFA Findings:**
   - Diffuse leakage in the macula of both eyes

Clinician’s treatment plan:

Eye	Clinician’s single best response for DME management	Clinician’s single best treatment response for ocular co-morbidity management:
RE		
LE		

Case Scenario 11:

1) **Patient Demographics:**
   - Age: 35 years
   - Gender: Female

2) **Diabetes Details:**
   - Type: Type 1 diabetes
   - Duration: 15 years
   - Control: HbA1c 6.8% on insulin pump therapy

3) **Systemic Co-morbidities:**
   - Renal Disease: No evidence of renal dysfunction
   - Hypertension: Well-controlled with ACE inhibitor
   - Anemia: Mild, managed with oral iron supplementation
   - Cardiovascular Disease: No history
   - Lipid Profile: Within normal limits

4) **Pregnancy Status:**
   - Currently in the second trimester of pregnancy

5) **Visual Symptoms:**
   - No recent onset visual symptoms

6) **Visual Acuity:**
   - Right Eye: 20/20
   - Left Eye: 20/25

7) **Lens Status:**
   - No cataract, pseudophakia, or aphakia

8) **Glaucoma Status:**
   - No pre-existing glaucoma

9) **Previous Treatment:**
   - No previous ocular interventions

10) **Fundus Findings:**
    - Mild non-proliferative diabetic retinopathy (NPDR) in both eyes with no macular edema

11) **OCT Findings:**
    - No evidence of macular edema in either eye

12) **FFA Findings:**
    - No abnormal macular findings on fluorescein angiography

Clinician’s treatment plan:

Eye	Clinician’s single best response for DME management	Clinician’s single best treatment response for ocular co-morbidity management:
RE		
LE		

Case Scenario 12:

1) **Patient Demographics:**
   - Age: 58 years
   - Gender: Male

2) **Diabetes Details:**
   - Type: Type 2 diabetes
   - Duration: 22 years
   - Control: HbA1c 8.5% on oral hypoglycemic agents

3) **Systemic Co-morbidities:**
   - Renal Disease: Moderate proteinuria
   - Hypertension: Poorly controlled despite multiple antihypertensive medications
   - Anemia: Moderate, managed with erythropoietin therapy
   - Cardiovascular Disease: History of stroke, on anticoagulation
   - Lipid Profile: Elevated LDL, managed with statin

4) **Pregnancy Status:**
   - Not applicable (male patient)

5) **Visual Symptoms:**
   - Gradual onset of distorted central vision

6) **Visual Acuity:**
   - Right Eye: 20/40
   - Left Eye: 20/30

7) **Lens Status:**
   - Early cataract in both eyes

8) **Glaucoma Status:**
   - No pre-existing glaucoma

9) **Previous Treatment:**
   - Previous focal laser therapy for diabetic macular edema in the right eye

10) **Fundus Findings:**
    - Moderate non-proliferative diabetic retinopathy (NPDR) in both eyes with center-involving macular edema in the right eye

11) **OCT Findings:**
    - Central macular thickness of 380 microns in the right eye; left eye has no macular edema

12) **FFA Findings:**
    - Focal leakage in the right eye indicating active macular edema

Clinician’s treatment plan:

Eye	Clinician’s single best response for DME management	Clinician’s single best treatment response for ocular co-morbidity management:
RE		
LE		

Case Scenario 13:

1) **Patient Demographics:**
   - Age: 45 years
   - Gender: Female

2) **Diabetes Details:**
   - Type: Type 2 diabetes
   - Duration: 10 years
   - Control: HbA1c 7.2% on oral hypoglycemic agents

3) **Systemic Co-morbidities:**
   - Renal Disease: Normal renal function
   - Hypertension: Well-controlled with ACE inhibitor
   - Anemia: No anemia
   - Cardiovascular Disease: No history
   - Lipid Profile: Within normal limits

4) **Pregnancy Status:**
   - Not applicable (postmenopausal)

5) **Visual Symptoms:**
   - Sudden onset of a shadow in the peripheral vision

6) **Visual Acuity:**
   - Right Eye: 20/20
   - Left Eye: 20/25

7) **Lens Status:**
   - No cataract, pseudophakia, or aphakia

8) **Glaucoma Status:**
   - No pre-existing glaucoma

9) **Previous Treatment:**
   - No previous ocular interventions

10) **Fundus Findings:**
   - Early signs of non-proliferative diabetic retinopathy (NPDR) in both eyes with no macular edema with posterior vitreous detachment

11) **OCT Findings:**
   - No evidence of macular edema in either eye

12) **FFA Findings:**
   - No abnormal findings on fluorescein angiography

Clinician's treatment plan:

Eye	Clinician's single best response for DME management	Clinician's single best treatment response for ocular co-morbidity management:
RE		
LE		

Case Scenario 14:

1) **Patient Demographics:**
   - Age: 50 years
   - Gender: Male

2) **Diabetes Details:**
   - Type: Type 2 diabetes
   - Duration: 18 years
   - Control: HbA1c 7.8% on oral hypoglycemic agents

3) **Systemic Co-morbidities:**
   - Renal Disease: Stage 2 chronic kidney disease
   - Hypertension: Well-controlled with angiotensin receptor blocker
   - Anemia: Mild, managed with oral iron supplementation
   - Cardiovascular Disease: No history
   - Lipid Profile: Elevated LDL, managed with statin

4) **Pregnancy Status:**
   - Not applicable (male patient)

5) **Visual Symptoms:**
   - No recent onset visual symptoms

6) **Visual Acuity:**
   - Right Eye: 20/30
   - Left Eye: 20/40

7) **Lens Status:**
   - Early cataract in both eyes

8) **Glaucoma Status:**
   - No pre-existing glaucoma

9) **Previous Treatment:**
   - History of pan retinal laser photocoagulation for proliferative diabetic retinopathy (PDR) in both eyes

10) **Fundus Findings:**
    - Stable PDR with laser scars in both eyes

11) **OCT Findings:**
    - No evidence of macular edema in either eye

12) **FFA Findings:**
    - No abnormal findings on fluorescein angiography

Clinician's treatment plan:

Eye	Clinician's single best response for DME management	Clinician's single best treatment response for ocular co-morbidity management:
RE		
LE		

Case Scenario 15:

1) **Patient Demographics:**
- Age: 60 years
- Gender: Female

2) **Diabetes Details:**
- Type: Type 2 diabetes
- Duration: 25 years
- Control: HbA1c 8.0% on insulin therapy

3) **Systemic Co-morbidities:**
- Renal Disease: Moderate proteinuria
- Hypertension: Poorly controlled despite multiple antihypertensive medications
- Anemia: Moderate, managed with erythropoietin therapy
- Cardiovascular Disease: History of heart failure since 3 months
- Lipid Profile: Elevated triglycerides and low HDL, difficult to manage

4) **Pregnancy Status:**
- Not applicable (postmenopausal)

5) **Visual Symptoms:**
- Blurring of vision

6) **Visual Acuity:**
- Right Eye: 20/25
- Left Eye: 20/30

7) **Lens Status:**
- Early cataract in both eyes

8) **Glaucoma Status:**
- No pre-existing glaucoma

9) **Previous Treatment:**
- Previous intravitreal anti-VEGF injections for diabetic macular edema in both eyes

10) **Fundus Findings:**
- Bilateral severe non-proliferative diabetic retinopathy (NPDR) with macular edema

11) **OCT Findings:**
- Center-involving macular edema in both eyes with a central macular thickness of 400 microns

12) **FFA Findings:**
- Diffuse leakage in the macula of both eyes

Clinician’s treatment plan:

Eye	Clinician’s single best response for DME management	Clinician’s single best treatment response for ocular co-morbidity management:
RE		
LE		

Case Scenario 16:

1) **Patient Demographics:**
- Age: 42 years
- Gender: Female

2) **Diabetes Details:**
- Type: Type 1 diabetes
- Duration: 20 years
- Control: HbA1c 7.5% on multiple daily injections

3) **Systemic Co-morbidities:**
- Renal Disease: No evidence of renal dysfunction
- Hypertension: Well-controlled with ACE inhibitor
- Anemia: Mild, no intervention required
- Cardiovascular Disease: No history
- Lipid Profile: Within normal limits

4) **Pregnancy Status:**
- Currently in the first trimester of pregnancy

5) **Visual Symptoms:**
- Sudden onset of visual distortion

6) **Visual Acuity:**
- Right Eye: 20/30
- Left Eye: 20/25

7) **Lens Status:**
- No cataract, pseudophakia, or aphakia

8) **Glaucoma Status:**
- No pre-existing glaucoma

9) **Previous Treatment:**
- No previous ocular interventions

10) **Fundus Findings:**
- Moderate non-proliferative diabetic retinopathy (NPDR) in both eyes with center-involving macular edema in the right eye

11) **OCT Findings:**
- Central macular thickness of 420 microns in the right eye; left eye has no macular edema

12) **FFA Findings:**
- Focal leakage in the right eye indicating active macular edema

Clinician's treatment plan:

Eye	Clinician's single best response for DME management	Clinician's single best treatment response for ocular co-morbidity management:
RE		
LE		

Case Scenario 17:

1) **Patient Demographics:**
   - Age: 55 years
   - Gender: Male

2) **Diabetes Details:**
   - Type: Type 2 diabetes
   - Duration: 15 years
   - Control: HbA1c 8.9% on insulin therapy

3) **Systemic Co-morbidities:**
   - Renal Disease: End-stage renal disease on hemodialysis
   - Hypertension: Poorly controlled despite multiple antihypertensive medications
   - Anemia: Severe, requiring erythropoietin therapy
   - Cardiovascular Disease: History of stroke, on anticoagulation
   - Lipid Profile: Elevated triglycerides and low HDL, difficult to manage

4) **Pregnancy Status:**
   - Not applicable (male patient)

5) **Visual Symptoms:**
   - Sudden onset of floaters and blurred vision

6) **Visual Acuity:**
   - Right Eye: 20/40
   - Left Eye: 20/30

7) **Lens Status:**
   - Pseudophakia in both eyes

8) **Glaucoma Status:**
   - History of narrow-angle glaucoma, well-controlled with laser peripheral iridotomy

9) **Previous Treatment:**
   - No previous ocular interventions

10) **Fundus Findings:**
    - Bilateral severe proliferative diabetic retinopathy (PDR) with vitreous hemorrhage in the right eye

11) **OCT Findings:**
    - No evidence of macular edema in either eye

12) **FFA Findings:**
    - Right eye shows diffuse leakage indicating active neovascularization

Clinician’s treatment plan:

Eye	Clinician’s single best response for DME management	Clinician’s single best treatment response for ocular co-morbidity management:
RE		
LE		

Case Scenario 18:

1) **Patient Demographics:**
   - Age: 50 years
   - Gender: Female

2) **Diabetes Details:**
   - Type: Type 2 diabetes
   - Duration: 25 years
   - Control: HbA1c 7.8% on oral hypoglycemic agents

3) **Systemic Co-morbidities:**
   - Renal Disease: Stage 3 chronic kidney disease
   - Hypertension: Well-controlled with angiotensin receptor blocker
   - Anemia: Moderate, managed with erythropoietin therapy
   - Cardiovascular Disease: History of coronary artery disease, on antiplatelet therapy
   - Lipid Profile: Elevated LDL, managed with statin

4) **Pregnancy Status:**
   - Not applicable (postmenopausal)

5) **Visual Symptoms:**
   - Gradual loss of peripheral vision

6) **Visual Acuity:**
   - Right Eye: 20/25
   - Left Eye: 20/30

7) **Lens Status:**
   - Early cataract in both eyes

8) **Glaucoma Status:**
   - No pre-existing glaucoma

9) **Previous Treatment:**
   - Previous focal laser therapy for diabetic macular edema in the left eye

10) **Fundus Findings:**
   - Moderate non-proliferative diabetic retinopathy (NPDR) in both eyes with no macular edema

11) **OCT Findings:**
   - No evidence of macular edema in either eye

12) **FFA Findings:**
   - No abnormal findings on fluorescein angiography

Clinician’s treatment plan:

Eye	Clinician’s single best response for DME management	Clinician’s single best treatment response for ocular co-morbidity management:
RE		
LE		

Case Scenario 19:

1) **Patient Demographics:**
   - Age: 68 years
   - Gender: Male

2) **Diabetes Details:**
   - Type: Type 2 diabetes
   - Duration: 30 years
   - Control: HbA1c 8.2% on insulin therapy

3) **Systemic Co-morbidities:**
   - Renal Disease: Moderate chronic kidney disease
   - Hypertension: Poorly controlled despite multiple antihypertensive medications
   - Anemia: Severe, requiring erythropoietin therapy
   - Cardiovascular Disease: History of heart failure
   - Lipid Profile: Elevated triglycerides and low HDL, difficult to manage

4) **Pregnancy Status:**
   - Not applicable (male patient)

5) **Visual Symptoms:**
   - Gradual onset of blurred vision

6) **Visual Acuity:**
   - Right Eye: 20/40
   - Left Eye: 20/30

7) **Lens Status:**
   - Early cataract in both eyes

8) **Glaucoma Status:**
   - No pre-existing glaucoma

9) **Previous Treatment:**
   - Previous pan retinal laser photocoagulation for proliferative diabetic retinopathy (PDR)1 month back in both eyes

10) **Fundus Findings:**
   - Stable PDR with laser scars and macular edema in both eyes

11) **OCT Findings:**
   - Both eyes show macular edema in both eyes with cystoid macular edema and neurosensory detachment

12) **FFA Findings:**
   - Diffuse macular leakage with diffuse optic disc staining

Clinician’s treatment plan:

Eye	Clinician’s single best response for DME management	Clinician’s single best treatment response for ocular co-morbidity management:
RE		
LE		

Case Scenario 20:

1) **Patient Demographics:**
   - Age: 55 years
   - Gender: Female

2) **Diabetes Details:**
   - Type: Type 2 diabetes
   - Duration: 18 years
   - Control: HbA1c 7.0% on oral hypoglycemic agents

3) **Systemic Co-morbidities:**
   - Renal Disease: Mild proteinuria
   - Hypertension: Well-controlled with ACE inhibitor
   - Anemia: Moderate, managed with oral iron supplementation
   - Cardiovascular Disease: History of myocardial infarction, on aspirin and statin
   - Lipid Profile: Within normal limits

4) **Pregnancy Status:**
   - Not applicable (postmenopausal)

5) **Visual Symptoms:**
   - Sudden onset of a shadow in the peripheral vision

6) **Visual Acuity:**
   - Right Eye: 20/20
   - Left Eye: 20/25

7) **Lens Status:**
   - No cataract, pseudophakia, or aphakia

8) **Glaucoma Status:**
   - No pre-existing glaucoma

9) **Previous Treatment:**
   - No previous ocular interventions

10) **Fundus Findings:**
    - Early signs of non-proliferative diabetic retinopathy (NPDR) in both eyes with no macular edema and presence of posterior vitreous detachment

11) **OCT Findings:**
    - No evidence of macular edema in either eye

12) **FFA Findings:**
    - No abnormal findings on fluorescein angiography

Clinician's treatment plan:

Eye	Clinician's single best response for DME management	Clinician's single best treatment response for ocular co-morbidity management:
RE		
LE		

Case Scenario 21:

1) **Patient Demographics:**
   - Age: 55 years
   - Gender: Female

2) **Diabetes Details:**
   - Type: Type 2 diabetes
   - Duration: 12 years
   - Control: HbA1c 6.5% on lifestyle modification

3) **Systemic Co-morbidities:**
   - Renal Disease: No evidence of renal dysfunction
   - Hypertension: Absent
   - Anemia: No anemia
   - Cardiovascular Disease: No history
   - Lipid Profile: Within normal limits

4) **Pregnancy Status:**
   - Not applicable (postmenopausal)

5) **Visual Symptoms:**
   - Recent onset of blurring in vision since 3 months

6) **Visual Acuity:**
   - Right Eye: 20/50
   - Left Eye: 20/30

7) **Lens Status:**
   - Early cataract in the right eye; pseudophakia in the left eye

8) **Glaucoma Status:**
   - No pre-existing glaucoma

9) **Previous Treatment:**
   - No h/o of any previous treatment

10) **Fundus Findings:**
    - Severe non-proliferative DR in both eyes with RE CSME and RE has inferior peripheral lattice degeneration

11) **OCT Findings:**
    - Center-involving DME in right eye with CSFT = 562 microns and left eye showing non-center involving DME

12) **FFA Findings:**
    - Both eyes show diffuse macular leakage

Clinician’s treatment plan:

Eye	Clinician’s single best response for DME management	Clinician’s single best treatment response for ocular co-morbidity management:
RE		
LE		

Case Scenario 22:

1) **Patient Demographics:**
   - Age: 65 years
   - Gender: Female

2) **Diabetes Details:**
   - Type: Type 2 diabetes
   - Duration: 12 years
   - Control: HbA1c 6.5% on lifestyle modification

3) **Systemic Co-morbidities:**
   - Renal Disease: No evidence of renal dysfunction
   - Hypertension: Absent
   - Anemia: No anemia
   - Cardiovascular Disease: No history
   - Lipid Profile: Within normal limits

4) **Pregnancy Status:**
   - Not applicable (postmenopausal)

5) **Visual Symptoms:**
   - Recent onset of blurring in vision since 3 months

6) **Visual Acuity:**
   - Right Eye: 20/50
   - Left Eye: 20/30

7) **Lens Status:**
   - Pseudophakia in both eyes

8) **Glaucoma Status:**
   - No pre-existing glaucoma

9) **Previous Treatment:**
   - past h/o right eye pars plana vitrectomy for dislocated nucleus during cataract surgery

10) **Fundus Findings:**
   - Severe non-proliferative DR in both eyes with RE CSME

11) **OCT Findings:**
   - Center-involving DME in right eye with CSFT = 562 microns and left eye showing non-center involving DME

12) **FFA Findings:**
   - Both eyes show diffuse macular leakage

Clinician's treatment plan:

Eye	Clinician's single best response for DME management	Clinician's single best treatment response for ocular co-morbidity management:
RE		
LE		

Case Scenario 23:

1) **Patient Demographics:**
   - Age: 55
   - Gender: Male

2) **Diabetes Details:**
   - Type: Type 2 Diabetes Mellitus
   - Duration: 15 years
   - Control: Sub-optimally controlled with HbA1c of 8.5%

3) **Systemic Co-morbidities:**
   - Renal Disease: Stage 2 chronic kidney disease
   - Hypertension: Controlled with medication
   - Anemia: Mild anemia
   - Cardiovascular Disease: History of myocardial infarction 5 years ago
   - Lipid Profile: Elevated triglycerides and LDL cholesterol

4) **Pregnancy Status:**
   - Not applicable (male patient)

5) **Visual Symptoms:**
   - Recent onset blurred vision, difficulty in reading

6) **Visual Acuity (Snellen's format):**
   - Right Eye: 20/40
   - Left Eye: 20/80

7) **Lens Status:**
   - Mild cataract in both eyes

8) **Pre-existing Glaucoma:**
   - None

9) **Treatment History:**
   - Previous focal laser treatment for macular edema in both eyes
   - Intravitreal anti-VEGF injections in the left eye 6 months ago

10) **Fundus Findings:**
   - Non-Proliferative Diabetic Retinopathy (NPDR) in right eye
   - Proliferative Diabetic Retinopathy (PDR) in the left eye

11) **OCT Findings:**
   - Center-involving macular edema in both eyes
   - Central Macular Thickness: Right Eye - 320 microns, Left Eye - 340 microns

12) **FFA Findings:**
   - Diffuse leakage in macula of both eyes
   - Macular ischemia in the left eye

Clinician’s treatment plan:

Eye	Clinician’s single best response for DME management	Clinician’s single best treatment response for ocular co-morbidity management:
RE		
LE		

Case Scenario 24:

1) **Patient Demographics:**
- Age: 28
- Gender: Female

2) **Diabetes Details:**
- Type: Gestational Diabetes Mellitus (during pregnancy)
- Duration: Diagnosed during the second trimester, now 6 months postpartum
- Control: Resolved after delivery

3) **Systemic Co-morbidities:**
- No significant co-morbidities

4) **Pregnancy Status:**
- Recent pregnancy with uncomplicated vaginal delivery

5) **Visual Symptoms:**
- Blurred vision since the last trimester of pregnancy

6) **Visual Acuity (Snellen's format):**
- Right Eye: 20/25
- Left Eye: 20/30

7) **Lens Status:**
- Clear lenses in both eyes

8) **Pre-existing Glaucoma:**
- None

9) **Treatment History:**
- No previous ocular treatments

10) **Fundus Findings:**
- Mild Non-Proliferative Diabetic Retinopathy (NPDR) in both eyes

11) **OCT Findings:**
- Non-center involving macular edema in both eyes
- Central Macular Thickness: Right Eye - 280 microns, Left Eye - 290 microns

12) **FFA Findings:**
- No significant leakage or macular ischemia observed

Clinician’s treatment plan:

Eye	Clinician’s single best response for DME management	Clinician’s single best treatment response for ocular co-morbidity management:
RE		
LE		

Case Scenario 25:

1. **Patient Demographics:**
   - Age: 55 years
   - Gender: Male

2. **Diabetes Details:**
   - Type: Type 2 Diabetes Mellitus
   - Duration: 10 years
   - Control: Fair, managed with oral hypoglycemic agents

3. **Systemic Co-morbidities:**
   - Absent

4. **Pregnancy (for female cases):**
   - Not applicable

5. **Visual Symptoms:**
   - Recent onset visual blurriness

6. **Visual Acuity (Snellen's format):**
   - Right Eye: 20/40
   - Left Eye: 20/30

7. **Lens Status:**
   - Pseudophakia in both eyes

8. **Pre-existing Glaucoma:**
   - Absent

9. **Previous Treatment History:**
   - Focal laser for macular edema in both eyes

10. **Fundus Findings:**
   - Non-Proliferative Diabetic Retinopathy (NPDR) with mild microaneurysms in both eyes

11. **OCT Findings:**
   - Location of Macular Edema: Center-involving edema in both eyes
   - Central Macular Thickness: Right Eye - 400 microns, Left Eye - 420 microns

12. **Fluorescein Angiography Findings:**
   - Diffuse leakage noted in macular region in both eyes

Clinician's treatment plan:

Eye	Clinician's single best response for DME management	Clinician's single best treatment response for ocular co-morbidity management:
RE		
LE		
